# Twitter in the Cross Fire—The Use of Social Media in the Westgate Mall Terror Attack in Kenya

**DOI:** 10.1371/journal.pone.0104136

**Published:** 2014-08-25

**Authors:** Tomer Simon, Avishay Goldberg, Limor Aharonson-Daniel, Dmitry Leykin, Bruria Adini

**Affiliations:** 1 Department of Emergency Medicine, Recanati School for Community Health Professions, Faculty of Health Sciences, Ben-Gurion University of the Negev, Beer Sheba, Israel; 2 PREPARED Center for Emergency Response Research, Ben-Gurion University of the Negev, Beer Sheba, Israel; 3 Ready.org.il – Emergency readiness and preparedness in Israel, Givatayim, Israel; 4 Department of Health Systems Management, Faculty of Health Sciences, Ben-Gurion University of the Negev, Beer Sheba, Israel; 5 Department of Psychology, Tel Hai Academic College, Kiryat Shmona, Israel; University of Pittsburgh Medical Center, United States of America

## Abstract

On September 2013 an attack on the Westgate mall in Kenya led to a four day siege, resulting in 67 fatalities and 175 wounded. During the crisis, Twitter became a crucial channel of communication between the government, emergency responders and the public, facilitating the emergency management of the event. The objectives of this paper are to present the main activities, use patterns and lessons learned from the use of the social media in the crisis. Using TwitterMate, a system developed to collect, store and analyze tweets, the main hashtags generated by the crowd and specific Twitter accounts of individuals, emergency responders and NGOs, were followed throughout the four day siege. A total of 67,849 tweets were collected and analyzed. Four main categories of hashtags were identified: geographical locations, terror attack, social support and organizations. The abundance of Twitter accounts providing official information made it difficult to synchronize and follow the flow of information. Many organizations posted simultaneously, by their manager and by the organization itself. Creating situational awareness was facilitated by information tweeted by the public. Threat assessment was updated through the information posted on social media. Security breaches led to the relay of sensitive data. At times, misinformation was only corrected after two days. Social media offer an accessible, widely available means for a bi-directional flow of information between the public and the authorities. In the crisis, all emergency responders used and leveraged social media networks for communicating both with the public and among themselves. A standard operating procedure should be developed to enable multiple responders to monitor, synchronize and integrate their social media feeds during emergencies. This will lead to better utilization and optimization of social media resources during crises, providing clear guidelines for communications and a hierarchy for dispersing information to the public and among responding organizations.

## Introduction

In recent years, the social media, and especially Twitter, have emerged as important, near real-time communication channels and technologies during emergency response. Social media consist of tools that enable open and online exchange of information through conversation, interaction and exchange of user generated content [1], [2]. These tools constantly produce vast amounts of data, both relevant and irrelevant. Researchers state that emergency managers should utilize filtering and pattern recognition algorithms on the data streams, in order to access important and meaningful information in real-time, facilitate an immediate response, and understand events as they unfold [3], [2]. Twitter has been shown to provide faster updates in developing emergencies in comparison to traditional media channels [4]. Social media, as opposed to traditional information and communications technologies, manage the content of the conversation or interaction as an artifact in the online environment (post or tweet) [5].

A recent study showed that Kenya is the second most active country in Africa on Twitter, surpassed only by South Africa [6]. Compared to the global average age of Twitter users, which is 39, 60% of Africa's users are 21 to 29, and 57% use Twitter from their mobile phones. Kenya's president, Uhuru Kenyatta, is the fourth most followed African leader [7].

The use of social media in emergencies, specifically Twitter and Facebook, is growing. The different social media channels have become an integral tool in the response of official authorities and the general public to emergency situations [2], [3], [8]. The continuous rise in Twitter and Facebook use during routine times and in emergencies indicates their potential contribution towards creation of situational awareness and status reports, tasks that have proven challenging in mass casualty incidents [4].

Sentiment analysis (also called subjectivity analysis or opinion mining) has been defined as the task of finding the opinions of authors about specific organizations [9]. It has been used extensively to gain insight into people's opinions, sentiments, evaluations, attitudes, and emotions from written language [10]. Nagy & Stamberger [11] noted that microblogs are an opportunity to search for critical information such as sentiments, and later use such information to rapidly detect the sentiment of the crowd towards crises or disasters. Kryvasheyeu, Chen, Moro, Hentenryck & Cebrian [12] studied sentiment analysis in over 50 million Twitter messages posted before, during and after Hurricane Sandy. They concluded that analyzing sentiments from Twitter (along with other metrics), may enable the use of “sentiment sensing” to detect and locate disasters. Previous studies have suggested that emergency dispatches and communications centers should convey information authoritatively and without emotion, even though they work in emotionally-charged environments, handling calls from victims, families of casualties and relaying information to and from field emergency personnel [13], [14]. Therefore we expect that the emotional orientation of communications dispatched by the operation centers will be less negative than the organizations' managers communicating from the scene.

In September 2013, an attack on the Westgate shopping mall in Kenya led to a four day siege, resulting in 67 fatalities and about 175 wounded. Around noon on September 21, an unknown number of armed terrorists entered the Westgate shopping mall in Nairobi, Kenya and took control. During the following days, the Kenyan security forces tried to retake the mall using ground and air forces. Kenyan emergency authorities managing the crisis established a perimeter surrounding the mall and provided support and medical treatment to the injured and their families. The Al-Shabaab militant group took responsibility for the attack through one of their Twitter accounts.

The Kenya Westgate Mall attack provides a unique opportunity to assess and understand how emergency organizations utilize social media to improve their responses to the event through content and activity analysis. This research aims to address three main hypotheses:

- Sentiment of emergency dispatches/EOC (Emergency Operations Center) tweets will be more positive than the sentiment of tweets made by the managers in the field.
- Use of social media during urban terror attacks leads to breaches of security.
- Emergency managers new to Twitter will use it as a basic, one-way, communications channel.

This case study offers two significant contributions. The first is a unique opportunity to study an event that took place in Africa, an understudied region in the field of social media. The second is the limited geographic nature of this event (which occurred in a defined set of buildings), enabling an understanding of the contribution of social media in an ongoing emergency. Currently, there is not enough evidence for best practice when incorporating social media in emergency response. In this study we make progress in understanding crisis communication patterns mediated by social media. Learning lessons from this event concerning the use of social media to create situational awareness can facilitate the creation of standard operating procedures for first responders that will encompass information sharing, control and command operations, and risk communication.

## Materials and Methods

### The Research Tools

The Kenya Westgate mall attack began on September 21, 2013 and lasted four days until September 24, 2013. Between September 21 and September 25, 2013, structured monitoring, collection, storing and analysis of Twitter information was carried out.

Data collection was performed using TwitterMate, a self-developed system specifically intended to collect tweets from Twitter. TwitterMate's architecture includes four main components- 1. A graphical user interface (GUI) where a user can define the basic Twitter search according to one or more usernames, hashtags and specific tweet IDs. TwitterMate can also collect the followers of specific Twitter users. Through the username search, TwitterMate is able to extract all tweets made by a specific Twitter account. The searches cover Twitter Search, REST and Stream APIs to collect tweets, and can run in an automatic or manual mode. 2, An Excel-based data store where all tweets are saved. Tweets are saved according their search parameters in different tabs, and are broken into 11 different information classifications. 3. A generic built-in API section that can operate and consume any natural language processing REST API calls i.e., Alchemy API. The API calls can be run on a specific tab in the Excel to analyze tweets. 4. In the Excel, further text analysis is conducted using a series of macros and formulas to extract hashtags and user accounts from the messages and clean tweets, running them through NLP analysis. The use of TwitterMate during a number of time intervals, as presented in Table 1, facilitated the collection of 67,849 tweets, based on the relevant hashtags and accounts of managers/organizations involved in the response to the event. Although TwitterMate collects tweets in all languages, only tweets in English, communicated by the emergency organizations were analyzed in this study, constituting 99.99% of all tweets made by the emergency organization accounts.

**Table 1:** TwitterMate tweets collection time intervals.

Date	Collection Time Interval
21/9/2013	13:39–18:44
22/9/2013	05:12–19:02
23/9/2013	05:07–15:01
24/9/2013	06:07–22:00
25/9/2013	05:56–09:51

As mentioned, TwitterMate has a built-in feature utilizing Alchemy API (http://www.alchemyapi.com/), a collection of natural language processing tools which provides multiple targeting options for sentiment analysis, including document-level, entity-level, and keyword-level directional, relational and user-specified sentiment analysis. In the present work, we used document-level (tweet level) sentiment analysis. Each tweet received a sentiment score: −1 (negative sentiment), 0 (neutral) and 1 (positive sentiment), and a general classification of polarity (negative, neutral or positive). Alchemy API has previously been used for sentiment analysis tasks in different contexts, including tourism [15], consumer behavior [16], education [17], [18], online technical support [19] and more. Alchemy API application on data extracted from Twitter has been documented as well [20]. Meehan et al. [15] examined the performance of a sentiment analysis classifier and reached 86.01% accuracy level (ACC). ACC represents the proportion of true results (true positives and true negatives) in the population. In the present study we randomly selected 15% of the total tweets and manually labeled them into three categories (negative, positive and neutral sentiment). We randomly selected 50% of managers' tweets, and 10% of organizations' tweets (total 299 tweets) and manually labeled them into sentiment categories: negative, positive and neutral. Overall, there was moderate agreement (61.5%) between two MA level researchers in labeling the tweets. A test set was created from the agreed upon tweets. Alchemy API sentiment analysis classification and our manual labeling were next entered into a confusion matrix to estimate true positives (manually classified as true, and classifier indicated true), false positives (manually classified as false, and classifier indicated true), false negatives (manually classified as true, and classifier indicated false) and true negatives (manually classified as false, and classifier indicated false). The ACC that was obtained was not satisfactory (61.27%) and it was found that both precision (i.e., proportion of true positives out of all true positives and false positives) and recall (i.e., sensitivity or the true positives out of all positives) were low for neutrality classification. After excluding all tweets that were classified neutral by Alchemy API (23%), we re-examined the performance of the classification on negative vs. positive values only (n = 87). This time we obtained acceptable ACC of 86.2%, with precision values of 90.48% and 84.85% for negative and positive (respectively), and recall values of 65.52% and 96.55% for negative and positive classifications (respectively). For our analysis, we used the sentiment score values (ranging from −1 to 1) for each tweet received, enabling us to incorporate more continues measure of sentiment. In addition, we used a binary measure of sentiment (negative vs. positive).

Twitter enables users to add a direct reference to a user in their tweets by inserting the user's account name (@username); i.e., a ‘mention’ [21] into the text. A mention can also be created by retweeting (“RT”) another person's message, and this user account is automatically inserted into the new message. When a person or organization is mentioned in a tweet, a notification is sent directly to their inbox. The Facebook pages of the governmental authorities, which can be publicly accessed, were analyzed in order to understand the differences in the use of various social media tools during emergencies and their effect on the public. For the sentiment analysis task, we first pre-processed the tweets by cleaning punctuation characters and other symbols frequently found in twitter, including: , : ; ? ( ) { } [ ] @ # $ & * + ∼ % and |. We also removed extra white spaces, converted website addresses (e.g. https://*) to URL, and converted @username to AT_USER. Finally, we removed stop words as they usually have little meaning. Such preprocessing is common and standard procedure in sentiment analysis [20], [22].

### Study Design

The event's hashtags, as well as specific users' and/or organizations' accounts were followed using TwitterMate. The collection process was initiated on September 21, based on the hashtag “#westgate”. Hashtag identification was based on content analysis and Excel formulas that extracted from each tweet any hashtag mentioned within it. The identified hashtags were then inserted manually into TwitterMate in order to integrate them in the automatic collection process. Two main hashtags (#WestGate and #WestGateAttack) and all governmental accounts and emergency organizations' accounts that participated in the response were followed and collected continually during the crisis, between September 21 and September 25. The participants followed were representatives of the Kenyan government and emergency authorities, and are thus considered formal and valid agents of Kenya's administration. Accordingly, tweets published by these organizations were considered as valuable and with impact on the population. We used the collected data to understand the progression and use of Twitter by the emergency authorities and to identify lessons learned. Using Statistical Package for the Social Sciences (SPSS), 19 we conducted a chi-squared test on the sentiment scores of the emergency authorities and their managers.

## Results

The collection was made during the hours of the day as it required manual interventions as new hashtags were created. All tweets made by the accounts mentioned in Table 2 from September 21 to September 25 were collected regardless of the time the collection process took place, as Twitter allows the collection of all tweets made by a specific account using its API.

**Table 2:** Extent of tweets, retweets and mentions during the Westgate mall attack.

User Name	Description	Twitter Join Date	Total # of Tweets	% (n) of RT's	% (n) of Mentions (excluding RT's)	% (n) of Tweets with Hashtags
@UKenyatta	President of The Republic of Kenya	26/8/2010	17	0% (0)	0% (0)	0% (0)
@WilliamsRuto	Deputy President of Kenya	12/7/2011	6	0% (0)	0% (0)	0% (0)
@PSCU_Digital	Home of Presidential and Government News	15/7/2013	228	25% (58)	54% (123)	78% (179)
@CabinetOfficeKE	Kenya's Cabinet Affairs Office in the Presidency	30/5/2013	78	95% (74)	0% (0)	24% (19)
@InteriorKE	Kenya's Ministry of Interior and Coordination of National Government	14/7/2013	1,523	49% (753)	26% (404)	32% (484)
@NDOCKenya	The Kenya National Disaster Operation Centre	28/4/2011	558	29% (163)	49% (274)	31% (172)
@joelenku	Cabinet Secretary - Interior and Co-ordination of National Government Kenya	4/9/2013	45	55% (25)	18% (8)	20% (9)
@F_Kimemia	Head of Civil Service and Secretary to the Cabinet of Kenya	31/5/2013	143	45% (65)	22% (32)	19% (28)
@IGKimaiyo	Inspector General of National Police Service, Kenya	24/6/2013	365	72% (264)	11% (40)	20% (75)
@KDFInfo	Kenya Defense Forces	13/9/2013	28	0% (0)	0% (0)	0% (0)
@MajorEChirchir	Kenya Military Spokesman	27/10/2011	35	28% (10)	46% (16)	6% (2)
@PoliceKE	Kenya Police	12/9/2013	569	54% (309)	21% (121)	24% (136)
@Abbas_Gullet	Secretary General of Kenya Red Cross	24/10/2011	18	55% (10)	22% (4)	55% (10)
@KenyaRedCross	Kenya Red Cross	30/4/2010	189	11% (22)	42% (79)	72% (136)
@AmbulanceKenya	St. John Ambulances	Not Available	81	34% (28)	18% (15)	62% (50)
@HSM_PR, @HSM_PRESOFFICE2, @hsmpress_	Al-Shabaab Terrorist Organization	Not available	258	1.5% (4)	6% (15)	89% (231)

### Sentiment Analysis

In order to avoid bias we used the AlchemyAPI to analyze only original tweets communicated by emergency organizations and their managers, while disregarding re-tweets.

The difference in sentiment average score between managers and organizations was statistically significant, as observed in an independent samples t-test, *t* (2004) = 1.76, *p*<0.05 (one-tailed), *d′* = 0.12. Managers' average sentiment (*M* = .04, *SD* = 0.17) was more positive than organizations' average sentiment (*M* = .01, *SD* = 0.18). However, using a two-sided chi-squared statistical test we found no difference in sentiment expression between managers and organizations, although descriptively, organizations had more negative classifications of their tweets (46.5% vs. 40.4%), while managers had more positive classifications of their tweets (59.6%). Therefore, the initial hypothesis that sentiments of emergency dispatches/EOC's tweets would be more positive than the sentiments of tweets communicated from on-site by the managers was refuted.

**Figure 1:**
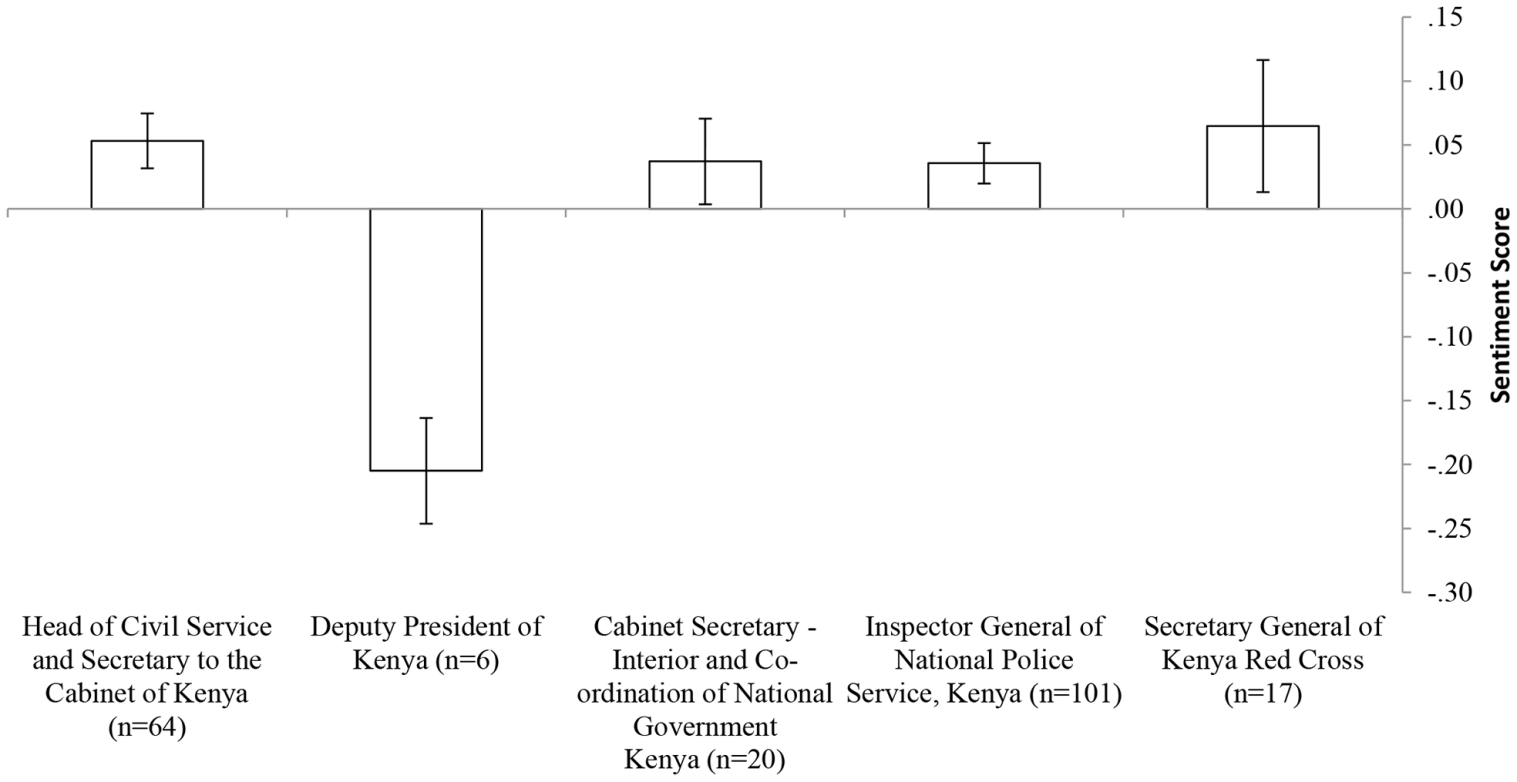
Average sentiment score of tweets made by the managers. For each account the number of tweets analyzed is provided (n).

Figure 1 presents a comparison of average sentiment scores of the managers communicating from the field. As can be seen, all managers' sentiment scores were positive (>0), except for tweets communicated by deputy president of Kenya William Ruto (n = 6) which were all analyzed as having a negative sentiment score.

**Figure 2:**
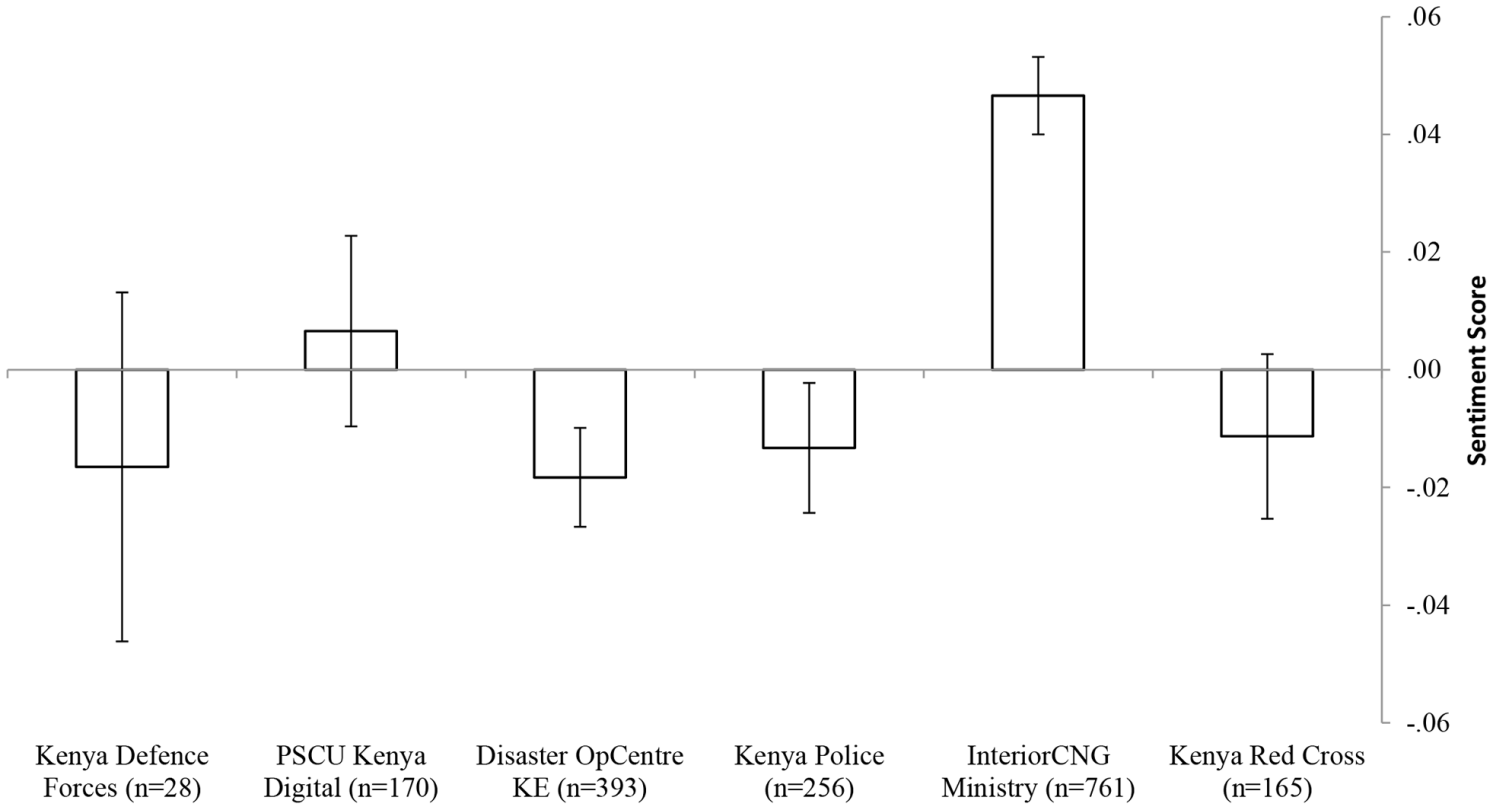
Average sentiment score of tweets made by the organizations. For each account the number of tweets analyzed is provided (n).

Figure 2 presents the average sentiment score of the emergency authorities responding to the event or coordinating the response to it. The average sentiments of the Ministry of the Interior as well as those of the PSCU Kenya Digital were positive compared to the average sentiment scores of the other emergency responders, which were negative.

### Hashtags

Through content analysis, four categories of hashtags used during the crisis were identified, referring to: 1) geographical locations – e.g., #WestGate, #WestGateMall, #Kenya, #Nairobi. These hashtags existed and were used on Twitter prior to the attack; 2) terror/violent attacks– e.g., #WestGateAttack, #WestGateSiege, #WestGateMallAttack and #WestGateShootout. Kenyan government officials, organizations and first responders used #WestGateAttack only; 3) social support, resilience and cohesiveness– e.g., #WeAreOne and #UnitedWeStand. The latter, though used by the public was absent from all official tweets; and, 4) organizations – e.g., #RedCross and #AlShabaab.

During the first days of the attack, numerous complaints were posted on Twitter stating that there were too many hashtags for the event or alternatively posting a list of hashtags, such as “One incident, many hashtags” or “So many different hashtags for today's tragedy #Westgate #WestgateMall #WestagateAttack #WestgateShootOut #WestGateMallShootOut”. Different variations were used inconsistently and interchangeably by the public, the media, and NGOs. On September 24 the PSCU Kenya Digital tweeted that all of the social media managers had held a synchronization meeting- “This morning we held a WhatsApp meeting with all bloggers running the @InteriorKE @PoliceKE @kdfinfo @NDOCKenya our voice is #WithOneAccord”, following which all responders consistently used the #WithOneAccord hashtag.

Moreover, while the fourth category of hashtags was present in many tweets, government officials preferred to use the “@” handle to mention the different organizations and individuals in their messages, as shown in Table 3.

**Table 3:** Government officials use of “@” in their tweets.

Official Account	# of Tweets	# of Tweets with “@”	Total %
Inspector General of the Kenya Police (@IGKimaiyo)	365	304	83.28%
Secretary of the Cabinet (@f_kimemia)	129	94	72.86%
Secretary of the Interior (@joelenku)	45	33	73.33%

### Mentions

Mentioning was frequently used by government officials and responding organizations as presented in Table 2. Both public and official users directly mentioned other official accounts during the entire crisis.

### Use of Twitter and Facebook

During this crisis, Twitter seems to have been the dominant social media platform used by the public and responding organizations. Though limited in scope, Facebook too was used. For example, the Kenya Police started posting on Twitter on September 21, but posted for the first time on its Facebook wall only on September 23, following an announcement on Twitter that included their Facebook page address [23]. Furthermore, the Kenya Police tweeted 569 times on Twitter during the entire crisis, while posting only ten times on Facebook. The Kenya Police had 20,267 followers on Twitter but only 2,506 fans (‘likes’) on Facebook. The same messages that were posted on Twitter were published on Facebook, keeping the hashtags while removing the mentions. Kenya's Ministry of Interior used its Twitter accounts 1,533 times while not posting a single message on their Facebook page.

### Participants

As an integral component of responding to the crisis, a number of governmental officials, organizations, first responders, and NGOs tweeted information concerning the event. The different organizations are presented in Table 2. The table also presents the date the Twitter account of the person/organization was created. The creation date of the Twitter account was extracted using the Twopcharts website (http://twopcharts.com/howlongontwitter). Eight of the accounts were created between May and September 2013, two of which (Kenya Police and Kenya Military) joined Twitter less than ten days prior to the attack.

In most organizations, communications were transmitted simultaneously by both the manager of the organization and the organization itself (see Table 4). Frequently, the managers of emergency organizations/ministries posted from the scene of the event, while coordinating the response, whereas the organization published more extensive information to the general public. For example, this was well displayed by the Ministry of Interior (@InteriorKE) and its minister Joseph Ole Lenku (@JoeLenku) communicating from the scene near the Westgate mall. Furthermore, both the Kenya Police and the Ministry of Interior tweeted throughout the event that official communications would be posted through their respective managers' Twitter accounts.

**Table 4:** Organizations or managers of organizations that tweeted during the Westgate mall attack.

Organization	Manager
Kenya Police (@PoliceKE)	Inspector General of National Police Service, Kenya (@IGKimayio)
Ministry of Interior (@InteriorKE)	Cabinet Secretary - Interior and Co-ordination of National Government Kenya (@JoeLenku)
Home of Presidential and Government News (@PSCU_Digital)	President of The Republic of Kenya (@UKenyatta)
Kenya Red Cross (@KenyaRedCross)	Secretary General of Kenya Red Cross (@Abbas_Gullet)
Cabinet Affairs Office in the Presidency (@CabinetOfficeKE)	Head of Civil Service and Secretary to the Cabinet of Kenya (@F_Kimemia)
Kenya Defense Forces (@KFDInfo)	Kenya Military Spokesman (@MajorEChirchir)

The abundance of Twitter accounts providing official information regarding the event made it difficult to synchronize and follow the flow of information. For example, on September 22, the Kenya Police stated “For systematic flow of information on the operation at West Gate please follow @kdfinfo @InteriorKE @PoliceKE @IGkimaiyo @joelenku”, which was retweeted 108 times. On September 23, the Ministry of Interior requested the Kenyan Military to follow them back on Twitter [24].

On the previous night, starting at 10:10 PM all government organizations started using the #WithOneAccord hashtag, initiated by the PSCU Kenya Digital account. On the following morning, September 24^th^, The PSCU Kenya Digital held a WhatsApp synchronization meeting with all of the social media managers in charge of the governmental Twitter and Facebook accounts [25]. Following the meeting, government organizations started using the #WithOneAccord hashtag consistently.

One first responder organization that participated in the response phase and which was referred to in other organizations' tweets was the Red Cross ambulance services (@EMS_Kenya). Prior to the attack, their account had been active on Twitter.

**Figure 3:**
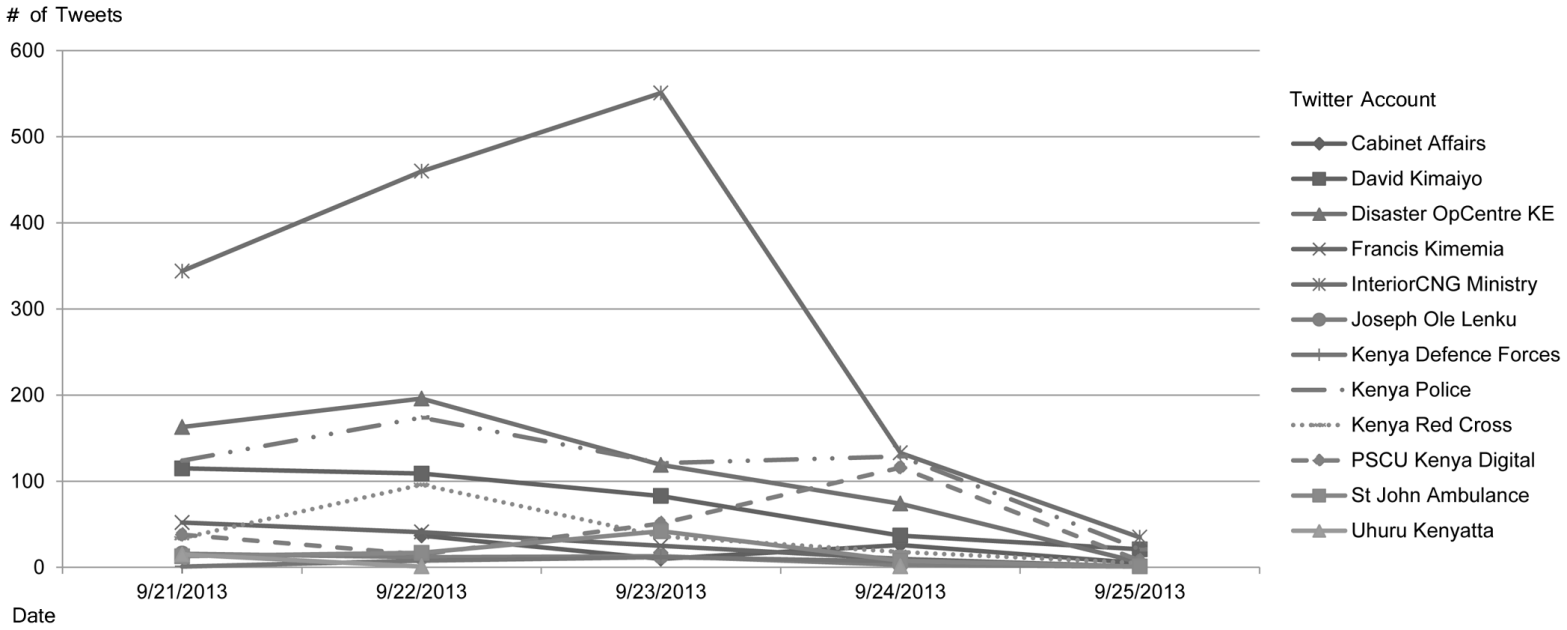
Number of tweets per day made by Twitter accounts of managers and organizations.

Figure 3 shows the number of tweets made by 12 Twitter accounts of government officials, organizations and NGOs during the four days of the crisis. The Kenyan Ministry of Interior tweeted between twice to five times more than other organizations, probably because it was the ministry in charge of coordination and response. The rise in the number of tweets made by the PSCU Kenya Digital, the official communications channel of the Kenyan government, can be explained by the efforts made to synchronize and align the response and messages to the public.

### Public

The general public was active on Twitter throughout the crisis. This activity can be classified into six main categories, 1) producing information concerning the event, 2) sharing and consolidating information, 3) expressing emotional and social support for individuals, communities or responders affected by the crisis 4) sharing concerns about privacy and security issues regarding social media, 5) requesting or offering to volunteer, and 6) criticizing the government and its security services.

### Terrorist organization

The Al-Shabaab organization twitted 258 times between September 21 and September 25. They claimed responsibility for the attack on the Westgate mall through Twitter. The Al-Shabbab used a number of accounts to try to avoid being caught by Twitter, but often had to change accounts as Twitter suspended them for breaching their terms of use. We followed three different accounts that were active on different dates “@hsmpress_” (21–22/9), “@HSM_PRESOFFICE2” (22–23/9) and “@HSM_PR” (24–25/9). The Al-Shabaab tweeted from the “@hsmpress_” account regarding their suspension on September 22 – “Alshabab has suffered another great casualty: Twitter account loss!”.

### Situational awareness updates by the first responders and government officials

Using their Twitter accounts, all government organizations and officials provided the public and other responding organizations with situational awareness updates. These updates included five classifications of information, including 1) risk communication and public warnings – e.g., Kenyan Police tweeted an hour after the attack: “The public should stay away from West Gate and its environs and we ask the media to refrain from any unnecessary comments.” The Kenya military tweeted: “Report any suspicious looking individuals or activities to the nearest security organizations of the Govt”. 2) Status updates about the armed response towards controlling the mall – e.g., the Kenya Police tweeted: “We have taken control of the ground floor and we urge you to be patient”, or the Kenya military, which communicated that: “KDF has dominated all floors of Westgate Mall building. Troops are now concentrating on clearing the building”. 3) Information hubs for other organizations and officials. Table 2 shows the percentage of retweets made by these accounts. 4) Communications and rumor control – e.g., the PSCU Kenya Digital tweeted: “Let us avoid unreliable sources in our Media and Social Media coverage of the Security Operation currently underway - Do not speculate.”, or the Minister of Interior that posted: “I urge Kenyans online to be responsible when you share information, it's better to share nothing and save a life.” 5) Reassurances, condolences and verbal reinforcements to the public and responding organizations– e.g., the Kenyan military tweeted on the second day: “All efforts are underway to bring this matter to a speedy conclusion”; the Minister of Interior tweeted: “I would like to reiterate that our security forces are fully in charge of the situation.”; and Secretary of the Cabinet tweeted: “My heartfelt condolences to the families and friends of those who perished in the heinous crime.”

### By the public

The public contributed to the situational awareness by tweeting information regarding the developments as they experienced them from the surroundings of the Westgate mall. Some even shared tweets with photos taken from the scene.

### Two-way communication and engagement

All responding organizations used Twitter to communicate and notify other organizations during the crisis. This was done by adding a mention to a tweet with a “cc” before it (as in emails), i.e., a tweet sent out by the Ministry of Interior: “Give way to ambulances making their way to #WestgateMall cc @NDOCKenya”. This tweet was sent out to the general public, but it also notified the Kenyan Disaster Operation Center by mentioning it, which retweeted it approximately two hours later. Almost an hour and a half after the attack began, the Minister of Interior arrived at the scene and tweeted: “I am at the scene and urge the public to be calm, give support to @IGkimaiyo and @PoliceKE and we will resolve this quickly. cc @InteriorKE”. It was retweeted by the Ministry of Interior less than a minute later.

The government organizations and officials were reading tweets that mentioned their names. In some cases they retweeted the original message, as the Police Inspector General retweeted: “RT @CitizenTVNews: Inspector General of Police David Kimaiyo via his twitter account @IGkimaiyo says the Westgate mall is currently surroun…”. The hypothesis that emergency organizations and managers new to Twitter would use it as a basic, one-way, communications channel was refuted. No differences were found in the use patterns of Twitter between new versus more experienced users.

### Crowdsourcing

We identified two ways emergency authorities requested assistance from the public during the crisis: 1) a direct call for information – e.g., the Inspector General of the police requested information regarding potential hostages “Kindly share information of the families who have been shopping and have not been traced. “ 2) Requesting the public to retweet their messages or share information on Facebook, e.g., the Kenya police tweeted “Forensic probe is underway. We urge the public to stay away for their own safety. Pls RT.”

### Threat assessment

At the beginning of the crisis, the responding organizations and the public did not understand the nature of the event. Stemming from the initial tweets, the police perceived that robbers rather than terrorists were attacking the mall. The Ministry of Interior tweeted approximately 30 minutes after the attack: “We've managed to evacuate some people to safety as @PoliceKE pursues the thugs”. Fifteen minutes later, they tweeted: “The IGP @IGkimaiyo is heading to the scene of crime to assess the situation. He'll also give a statement.” Approximately three hours after the attack began, Reuters tweeted that there was a possibility that terrorists might be perpetrating the attack.

### Operational security and censorship

Both the responding organizations and the public were wary about operational security. Many bystanders and journalists on site tweeted and broadcast their operations in real time. One bystander tweeted “why is this our media showing us the cops sneaking in??.” The Kenyan military tweeted “We urge all Kenyans to be cautious on what is being communicated on social media.”, and the PSCU Digital Kenya tweeted “We are appealing to Media to avoid showing photos of our @kdfinfo soldiers. Kindly, Only tweet what you are absolutely sure about #Westgate.” In one instance the Kenya Police asked a Twitter user to delete a message that contained pictures of military helicopters preparing to launch an attack on the mall [26]. On the second day of the crisis, the Kenyan Disaster Operation Center tweeted to a news channel asking that they remove a story “@CapitalFM_kenya Please take that story down. It is misleading and bound to confuse.” Eight minutes later they tweeted “Once again we request media houses to exercise caution in airing/publishing stories on operations at Westgate which are still sensitive.” These requests to the media and the public continued throughout the entire crisis. The Ministry of the Interior tweeted on the morning of the second day to a specific user “kindly keep such thoughts or DM them. The enemy is somewhere watching” (DM refers to a private direct message on Twitter). The hypothesis that the use of social media during urban terror attacks leads to breaches of security was confirmed.

On the third day of the crisis, the police published a direct threat of prosecution to those sharing ‘repugnant’ information on Twitter - “Those who have shared content that is repugnant to our sense of nationhood be warned that you will be found and charged.”

### Misinformation

During the first day of the attack, two unique tweets were posted, claiming to show pictures of the attackers. These messages were retweeted 106 times, 81 of them within 30 minutes. We found that these pictures were not of the attackers but rather of the Kenyan armed forces. The photos were removed after approximately two days.

### Geo-location information

Tweets can include the coordinates of the location the tweet was sent from. While government organizations and officials' accounts did not include geo-location information, 0.9% of tweets collected from the public included geo-location information. Out of all the accounts listed in Table 2, geo-location information was identified in only two tweets of Deputy President Ruto, placing him directly next to the Westgate mall.

The location of the managers was deduced based on the tweets' text or geo-location information. Although they did not include their own location they did include location based information in the text of the messages, whether with clearly identifiable information or with a location reference. One example of a tweet with a clear location can be found in a tweet by the Kenyan Disaster Operation Center 20 minutes after the attack had begun “Mwanzi Road between Peponi Rd and Ring Road Parklands is CLOSED TO ALL TRAFFIC.” The Ministry of Interior tweeted a message that included location based information by referring to an identifiable place “Roads adjacent to the #WestGateMall have been cordoned off, PLEASE use alternative route.”

### Verified Twitter accounts

On the third day of the attack, Twitter contacted the Kenyan government so that they could verify their accounts used to publish information on the Westgate attack. PSCU Kenya Digital first tweeted about it “@twitter has shared its support by contacting @PSCU_Digital to verify Government accounts-we have started with those communicating #WestGate.” In the following three hours, most accounts of the Kenyan government organizations and officials, including the Kenya Red Cross, were verified.

Prior to the verification process, the governmental organizations had to inform the public as to which accounts were sharing verified information, as tweeted by the Kenya Police on the second day “We urge all Kenyans to wait for official information from @InteriorKE @IGkimaiyo @joelenku @StateHouseKenya.”

## Discussion

Our findings show that Twitter served as an integral tool for emergency management in Kenya; all emergency related organizations and officials had accounts and actively communicated through them during the crisis, both with the public and among themselves. The use of Twitter in Kenya is not restricted to emergency situations alone but rather characterizes routine activities throughout the year. Merchant et al. [27] have presented the advantages of integrating social media into routine activities of emergency organizations.

The response to the attack was managed and coordinated by the Kenyan Police, supported by other first responders including the Red Cross ambulance service, EMS, and the fire brigade. The Kenyan military arrived on the first day to support and enhance the response, and the Kenyan National Disaster Operations Center oversaw the entire operation.

As presented in previous emergencies by different governments worldwide, the Kenyan government and ministers widely adopted the use of the social media during the terror attack and leveraged it in order to better understand the situation in real time, communicate with the public and improve their safety [2], [28]. The responding organizations often used two voices to communicate, both through the account of the organization and through the account of its manager.

Service providers, and especially public officials, emergency responders and spokespersons [13], are expected to manage their feelings while working with the public (control their “emotional labor”) and even “suppress their own feelings in order to show desirable work-related emotions” [29]. As described by Guy et al. [13] emergency responders are trained to “keep their emotions in check” and provide compassionate and emphatic service to those in need during emergencies, even though the surroundings may be chaotic and intense. Aligned with these expectations, we found that managers of the responding authorities tended to be more positive in their communications with the public. The initial hypothesis that emergency dispatch operators, working in a more disconnected environment than their on-site managers, would present more positive sentiment was refuted. Their communications tended to be more negative than those of their managers. This finding contrasts with former studies recommending that emergency dispatches and communications centers minimize the use of emotion during their communication with the public despite their emotionally charged work environment [13], [14]. However, emergency managers and public officials using positive language in their communications with the public during a crisis contributed to increased trust and credibility. The high engagement of the managers on Twitter throughout the crisis can be explained by the fact that they were considered more “approachable” by the public as they used positive language in their messages. In his paper, Seeger [30] showed that spokespersons using compassion and empathy in their communications enhanced their credibility and legitimacy in the eyes of the public, who would also respond more favorably during the crisis, Twitter's 140 characters limitation made managers write simple messages, which were more easily understood by the general public and could be repeated. This is consistent with the findings of Wray et al. [31], indicating that messages in emergencies should use basic and positive language throughout the crisis to provide clear information. During this crisis senior managers and government officials communicated from the scene also providing eyewitness accounts, which we think enhanced their public image and credibility. Messages from high ranking officials, or experts, are perceived by the public as credible sources of information, as presented by Reynolds et al. [32].

In this study, we found 15 different Twitter accounts disseminating and communicating messages, which caused information overload and synchronization problems. Bharosa, et al. [33] showed that information sharing between organizations during emergency response is one the biggest challenges. This may indicate that although Twitter has been implemented as a formal communication channel and organizations are proficient users, a standard operating procedure for its synchronization and use during emergency situations has not yet been formulated and formalized.

Official responders and organizations were not prepared to manage Twitter communications 24/7 during the crisis, and had long breaks during the night, sometimes for up to 10 hours. It was also apparent that some organizations did not know, or were unaware of the Twitter accounts of other responding organizations, creating gaps between the ministry and the military. In one case we found that the Ministry of Interior was unaware of the Kenyan Military's Twitter account, although it was active from the first day of the crisis. Most probably, this resulted from the Military tweeting messages without any hashtag or mentions, causing them to be visible only to their direct followers.

The rapid rise in information available on Twitter within minutes after the initiation of the attack may be explained by two main factors: Twitter is highly used in Kenya and thus serves as a popular platform for sharing information [6], and the time and place of the terror attack, a central shopping mall in the capital, on a weekend, when it was crowded and active. During the crisis, individuals strived to connect with their communities and country, and the social media facilitated this quick and broad bonding. Furthermore, Twitter enabled the public to get the information in almost real-time and with very easy access as the crisis developed. This strengthens the use patterns identified in previous studies regarding the use of social media by the public in emergencies [34], [35], [8].

Throughout the crisis civilians, reporters, and responding organizations, as well as the terrorists, shared information on Twitter about the status of the response. From the tweets it was clear that many media personnel were present in the vicinity of the mall, as were numerous bystanders. During emergencies it is common to access situational awareness updates from the responding organizations, the media and the general public on Twitter [36], [37]. In the present attack, reporters and bystanders shared information, both textual and visual, in almost real-time, with no control and with minimal official monitoring and censorship, regarding the status and location of the forces. This kind of information may endanger the lives of the responders, and might have contributed to the prolonged siege, as the terrorists received live information detailing the armed response against them and were able to use this information to enhance their response. A study on the 2008 Mumbai terror attack suggests that “unregulated real time Twitter postings can contribute to increase the level of situation awareness for terrorist groups to make their attack decision” [38]. This indeed is a major concern that should be considered in future events. It presents a challenge, as censorship and regulation are alien to the nature of social media.

From this crisis we can learn that the ‘traditional’ two-way communications channel view of Twitter was used with a more extensive approach, as a cross-hierarchy, inter- and intra-organizational tool for communications and coordination of the response. All responding entities and their managers, whether ministers, managers or commanders, used it to communicate with the public, with their own organization and with other responding organizations. This indicates that the organizations have the support of their management to implement and use Twitter, but nevertheless, a standard operating procedure (SOP) is crucially needed for effective implementation in emergencies. The SOP should delineate ways to synchronize communication between official first responders and emergency organizations, such as the WhatsApp synchronization meeting that was conducted during the Kenyan terror attack. This online meeting included all bloggers managing the social media accounts of the government organizations, emphasizing the fact that the social media managers are not employees of the government. The SOP should also relate to the challenge of continuous communication during crises. The fact that during the night there was a pause in Twitter updates to the public, sometimes for up to ten hours, suggests that only one person operated the social media account, lacking replacements or organized shifts. Kenya's Police Twitter operator reported this in one of his tweets referring the public to other official accounts for updates – “After almost 36 hours without sleep, I'm off duty at Visa Oshwal Center. Please follow @kpsya @interiorke @policeke @kdfinfo.via @kangai” [39]. Kavanaugh et al. [2] stated that a government organization that wants to employ a social media tool for crisis communications must first define the policies, as well as outline what information should be shared and through which channels. As we found, the Kenya government operates at least three social media networks (Twitter, Facebook and WhatsApp), with unclear strategy as to what information should be published on which network and time frames. Latonero & Shklovski [40] recommended incorporating a public information officer, or technology evangelists in the organization.

Publishing situational awareness updates through social media during emergencies is a common practice for numerous participants [37]. Although emergency organizations were highly active on Twitter during the attack, they provided only minimal and general situational awareness updates to the public. More specific situational updates were published on Twitter by the public and journalists as they witnessed and reported it, while emergency organizations continuously published their dissatisfaction with this practice. We found that during routine times these same organizations published situational updates through Twitter. This contrast probably led to confusion of the population. The need to clearly define expectations of both the authorities and the public, as well as direct policies of utilization of the social media in response to emergencies, reinforces the need to develop an SOP that will provide tools and guidelines for when, how and what can be made public during emergencies. Furthermore, such an SOP could suggest ways for emergency managers in the field to coordinate their messages and status among themselves and with the emergency operation centers. Twitter and other social media could be used to support public and open communications, but could also convey private messages that are not accessible or visible to unwanted parties. This method of operation can serve to reduce the need for information coordination meetings, increasing managers' exposure to data from the field as well as freeing valuable time that could be dedicated to putting more focus on managing the crisis.

During the first day of the attack, many civilians published photos of what they thought were the attackers. Some of these photos were retweeted by hundreds, not knowing that they were incorrect. The same photos were published by news agencies, such as the New York Times, describing them as “plainclothes officers” [41]. Publishing misinformation may occur during emergencies, especially during the initial phases of the response when the situation is unclear and characterized by speculation. While analyzing the tweets we found that these images were often deleted by the users, most probably because they realized their mistake. This indicates that social media can be ‘self-regulating’ to filter and refute misinformation, and even rumors, as described by Mendoza et al. [42]. We did not find the Kenyan authorities publishing corrections to disinformation or to rumors during the crisis.

The Kenyan officials and organizations tried to direct the public to their accounts for access to verified and reliable information, but this was not necessarily implemented. This reflects two main challenges of using social media in emergencies: Anyone can open an account on social media sites and publish information [27], and during emergency situations people are exposed to large amounts of information that might contain inaccuracies [43]. Twitter, and other social media sites, understand the need to verify the accounts of official emergency authorities, and Twitter has recently released a new tool to allow sending verified emergency notifications, called Twitter Alerts, directly to users' phones [44].

## Conclusions

Social media offer an accessible, widely available means for bi-directional flow of information between the public and the authorities in charge of public safety. Their use is growing across geographies, cultures, languages and organizations. This manuscript portrays the use of social media by the various participants in an unexpected event, and focuses mainly on use by emergency organizations. Emergency management almost always entails the response of multiple organizations and NGOs; in this crisis, all of these used and leveraged social media networks, not solely for communicating with the public, but also among themselves, frequently using two different Twitter accounts (for managers and for organizations), and for both public and private communications. Twitter offers emergency entities a communications platform that is ubiquitous and does not have interoperability issues, like other communication systems. This event showed that such a scenario may cause synchronization and coordination problems affecting response, and may waste valuable resources. Lessons learned from this event should contribute to the development of an SOP that will allow multiple responders to monitor, synchronize and integrate their social media feeds during emergencies. This will lead to better utilization and optimization of social media resources during crises, providing clear guidelines for communications and a hierarchy for dispersing information to the public and responding organizations during emergency situations. We recommend that future research analyze the differences between routine and emergency utilization of the social media by emergency organizations.

Limitations of the present study include the current state of sentiment analysis of crisis communication. Unfortunately, there is still a lack of studies analyzing emotional valence of micro-blog information during emergencies [12], and more work is needed to adapt sentiment analysis to emergency situations. During emergency situations some communications behavior, as seen in the present study, include words like “terrorists”, or “attack”, which are part of the emergency event (e.g., the Westgate mall attack). These terms should be analyzed as neutral words as they are informative pieces of information and do not necessarily reflect an opinion or an attitude of the communicator.

TwitterMate or similar instruments can become a tool that will help to manage and coordinate emergency events. Governments as well as emergency organizations should consider the availability of social media tools when they define their policies, and outline information they wish to share, and through what channels. Regulating the information provided by the public is yet another challenge that can possibly be better achieved through education rather than through regulation.
